# Implementation of family-centered care in Pediatric Gastroscopy

**DOI:** 10.12669/pjms.42.2.12891

**Published:** 2026-02

**Authors:** Tongtong Ma, Xiaoyu Song, Cuihong Zhao, Dawei Tian, Guoliang Zhang

**Affiliations:** 1 Tongtong Ma Department of Gastroenterology, Maternity & Child Care Center of Qinhuangdao, Qinhuangdao 066000, Hebei, China; 2 Xiaoyu Song Department of Gastroenterology, Maternity & Child Care Center of Qinhuangdao, Qinhuangdao 066000, Hebei, China; 3 Cuihong Zhao Department of Gastroenterology, Maternity & Child Care Center of Qinhuangdao, Qinhuangdao 066000, Hebei, China; 4 Dawei Tian Department of Gastroenterology, Maternity & Child Care Center of Qinhuangdao, Qinhuangdao 066000, Hebei, China; 5 Guoliang Zhang Department of Gastroenterology, Maternity & Child Care Center of Qinhuangdao, Qinhuangdao 066000, Hebei, China

**Keywords:** Family-centered care model, Gastroscopy, Pediatric gastropathy, Nursing intervention, Parental caregiving competence

## Abstract

**Objective:**

To evaluate the implementation of the family-centered care(FCC) model in pediatric patients undergoing diagnostic procedures for gastric disorders.

**Methodology:**

This retrospective study analyzed complete medical records of pediatric patients presenting with gastrointestinal symptoms and undergoing painless gastroscopy at Maternity & Child Care Center of Qinhuangdao between January 2024 to June 2025. Ninety children who received FCC and 90 who received standard care were included using concealed allocation and a single-blind design. Primary outcomes included procedural cooperation and duration, anxiety levels, physiological stress markers, parental knowledge scores, postoperative satisfaction at one-month follow-up, incidence of adverse events and recurrence of gastrointestinal symptoms.

**Results:**

The cooperation rate in the FCC group was significantly higher compared with the standard care group(*P<* 0.05). The procedural duration was also significantly shorter in the FCC group(*P<* 0.05). Baseline SAI and TAI scores did not differ significantly between groups(both *P>* 0.05); however, post-intervention scores were significantly lower in the FCC group, with more pronounced differences in SAI scores(*P<* 0.001). Increases in SBP, DBP and HR after the procedure were significantly greater in the standard care group than in the FCC group(all *P<* 0.001). One-month follow-up showed that the FCC group had significantly better scores in patient and caregiver satisfaction, lower incidence of adverse events and reduced recurrence of gastrointestinal symptoms (all *P<* 0.001).

**Conclusion:**

The use of the FCC model in pediatric gastroscopy may significantly enhance patient cooperation and procedural safety, reduce anxiety and physiological stress responses, strengthen parental caregiving competence and improve long-term outcomes.

## INTRODUCTION

The incidence of pediatric gastric disorders, such as functional dyspepsia and *Helicobacter pylori* (Hp) infection, has been steadily increasing in recent years. Gastroscopy, while considered the gold standard for diagnosing such conditions, is an invasive procedure that often provokes intense fear and resistance in children, potentially leading to procedural interruptions and heightened stress responses.^1^ Studies indicate that approximately 60% to 80% of pediatric patients experience moderate to severe preoperative anxiety, which significantly undermines the safety and efficiency of diagnostic and therapeutic interventions.^2^

Recent research, both domestically and internationally, has increasingly focused on non-pharmacological strategies to improve children’s healthcare experiences.^3^ In countries with advanced healthcare systems, the family-centered care (FCC) model has been widely adopted and practiced. This model emphasizes comprehensive parental empowerment, aiming to enhance parental engagement and competence throughout the medical care process. By doing so, FCC has been shown to improve children’s cooperation and adherence to various diagnostic and therapeutic procedures.^4^,^5^ In contrast, FCC practices in China remain in the exploratory stage. Existing interventions often rely on a limited model of perioperative parental accompaniment, lacking standardized training systems and multidimensional outcome evaluations.^6^

Furthermore, current implementations frequently fail to integrate education, skills training and psychological support. Assessment metrics tend to be narrow in scope, neglecting important factors such as physiological stress responses and long-term recovery quality. In many cases, parents remain passive participants and their caregiving capacity is insufficiently leveraged.^7^ In response to these gaps, this study designed an FCC intervention protocol and conducted a retrospective randomized controlled trial to evaluate its impact on procedural cooperation during gastroscopy, anxiety levels, physiological stress markers and parental knowledge. Additionally, postoperative adverse events and symptom recurrence were tracked at a one-month follow-up. The ultimate goal is to provide empirical evidence to support the development of standardized family engagement pathways and promote a more humane approach to pediatric gastrointestinal endoscopy.

## METHODOLOGY

This study adopted a retrospective research design, reviewing the complete medical records of pediatric patients who presented with gastrointestinal symptoms and underwent painless gastroscopy at Maternity & Child Care Center of Qinhuangdao between January 2024 to June 2025. Based on nursing documentation, ninety children who received FCC and 90 who received standard care were included. Concealed allocation was performed using sealed, opaque envelopes and a single-blind design was implemented. In the FCC group, there were 52 boys (57.8%) and 38 girls (42.2%), with a mean age of 7.81 ± 2.43 years, a mean disease duration of 6.53 ± 3.72 months and a mean body mass index (BMI) of 16.42 ± 1.91 kg/m^2^. In the standard care group, 46 were boys (51.1%) and 44 were girls (48.9%), with a mean age of 7.45 ± 2.29 years, a mean disease duration of 6.49 ± 3.81 months and a mean BMI of 16.09 ± 1.75 kg/m^2^. Statistical analysis revealed no significant differences between groups in baseline clinical characteristics, including sex distribution, age, disease duration and BMI (all *P >* 0.05).

### Ethical Approval:

The study was approved by the Institutional Ethics Committee of Maternity & Child Care Center of Qinhuangdao (No: QHDFY-20250600301; Date: June 03, 2025) and written informed consent was obtained from all *participants’ guardians*.

### Inclusion criteria:

- Aged 6-12 years old.
- Pediatric patients clinically assessed as requiring painless gastroscopy.
- Children who fulfilled the diagnostic criteria for Hp infection, Hp-positive patients diagnosed by endoscopic pathological staining and rapid urease test.
- Legal guardians who fully understood the study procedures and voluntarily signed a written informed consent form.
- Absence of contraindications to endoscopy, as confirmed by clinical evaluation, including exclusion of severe cardiopulmonary insufficiency.

### Exclusion criteria:

- History of severe adverse reactions during prior electronic gastroscopy, such as laryngospasm or bronchospasm.
- Presence of organic cognitive impairment or psychiatric disorders that would impair procedural cooperation.
- Use of central nervous system sedatives within 24 hours prior to the procedure.
- Incomplete clinical data, defined as missing more than 20% of key laboratory or imaging parameters.

### Standard Care Group:

According to the medical records, children in the standard care group received only standard preoperative preparation, procedural management and basic postoperative instructions. The standard protocol was as follows.

Children were instructed to fast strictly for four hours prior to the procedure. Anesthesia was induced using propofol at a dosage of 2- mg/kg body weight. Patients were positioned in the left lateral decubitus position and properly secured before the gastroscopy. For cases where endoscopic examination revealed diffuse gastric mucosal hyperemia and edema, along with characteristic sea urchin-like changes suggestive of Hp infection, two tissue biopsy samples were collected, one from the antrum and one from the body of the stomach, for a rapid urease test (RUT). Throughout the procedure, bispectral index (BIS) monitoring was used to continuously assess and adjust the depth of anesthesia in real time. Vital signs, including systolic blood pressure (SBP), diastolic blood pressure (DBP), heart rate (HR) and oxygen saturation (SpO_2_), were continuously monitored. If any intraoperative abnormalities occurred, immediate emergency response measures were initiated, including positive-pressure oxygen delivery via facemask, positional adjustments and prompt notification of the anesthesiologist. All procedures were performed in strict accordance with aseptic surgical protocols.

### FCC Group:

According to medical records, parents of children in the FCC group participated in a structured, hospital-implemented FCC program administered before and after the procedure. The core components of the intervention included a four-module training course led by the department’s head nurse:

### Educational Module:

Parents received instruction on gastric anatomy and the pathogenesis of Hp infection through 3D animation demonstrations, accompanied by the Children’s Gastric Health Handbook.

### Emergency Response Module:

Parents practiced simulated emergency management of vomiting using a manikin, with emphasis on the “golden 30-minute” intervention window to prevent aspiration and other complications.

### Soothing Techniques Module:

A scenario-based training zone was set up for parents to practice the “three-step soothing method,” which included reading picture books with the child, using stress-relief balls and awarding encouragement badges to promote emotional security and cooperation.

### Safety and Positioning Module:

Using a gastroscopy simulation model, parents were taught the “three-point fixation method” for positioning adjustments to ensure procedural safety and optimize the child’s cooperation. Within 24 hours postoperatively, the primary nurse provided each caregiver with a personalized recovery manual, which included a liquid diet plan and medication schedule tailored to the child’s body weight. On postoperative Day-3, a follow-up call was made by the same nurse, with a focus on evaluating the caregiver’s ability to recognize abnormal symptoms in the child. The telephone assessment covered key indicators such as the nature of vomitus and the specific location of abdominal pain.

### Outcome measures:

The number of children classified as cooperative or uncooperative was extracted from the nursing records. Cooperation was defined as the ability to complete the gastroscopy smoothly after being comforted by parents and guided by nursing staff; failure to complete the examination under these conditions was defined as noncooperation. Procedural duration (in minutes) for each child was retrieved from the anesthesia records and included the total duration for gastroscopy and, when applicable, the RUT.

The State-Trait Anxiety Inventory (STAI),^8^ including the State Anxiety Inventory (SAI) and Trait Anxiety Inventory (TAI), was used to assess the children’s anxiety levels. Each subscale contains 20 items scored on a 4-point Likert scale, yielding total scores ranging from 20 to 80. Higher scores indicate greater anxiety.

Physiological parameters were extracted from patient monitor records. Thirty minutes before and thirty minutes after the gastroscopy, systolic blood pressure (SBP), diastolic blood pressure (DBP) and heart rate (HR) were measured using a calibrated electronic sphygmomanometer (Omron HEM-7121, Omron Healthcare (China) Co., Ltd.) and a pulse oximeter (Masimo Rad-97, Masimo Corporation). All children were measured in the supine position. Readings were taken three times consecutively and the average value was used. In cases of poor cooperation, the upper limb was secured during measurement and crying-induced measurement bias was recorded.

A self-designed questionnaire was used to assess caregivers’ knowledge before and after nursing intervention. The questionnaire comprised four domains: pharmacological treatment, dietary management, complication prevention and disease monitoring. Each domain was scored out of 100, with higher scores indicating better understanding and caregiving competence.

Follow-up data collected one month after the procedure were used to compare satisfaction scores from children and their caregivers, the incidence of adverse events and the recurrence rate of gastrointestinal symptoms.

### Statistical analysis:

All statistical analyses were performed using SPSS 26.0. Continuous variables were expressed as mean ± standard deviation (*x̄±s*). Categorical variables were analyzed using the chi-square (χ^2^) test. Comparisons between groups were analyzed using independent samples *t*-tests. A *P*-value <0.05 was considered statistically significant.

## RESULTS

The number of children who cooperated during the pre-procedural phase of painless gastroscopy was significantly higher in the FCC group (82/90, 91.11%) compared with the standard care group (68/90, 75.56%) (*P <* 0.05). Furthermore, the time required to complete the examination was significantly shorter in the FCC group than in the standard care group (*P <* 0.05) Table-I.

**Table-I:** Procedural cooperation and duration in pediatric patients.

Group	n	Cooperation level (n[%])		Procedural duration (min)
		Cooperative	Uncooperative	
FCC	90	82(91.11)	8(8.89)	31.88±2.63
Standard care	90	68(75.56)	22(24.44)	46.94±1.99
χ^2^/t-value		7.840		43.320
P-value		0.005		<0.001

Baseline SAI and TAI scores did not differ significantly between groups (both *P >* 0.05); however, post-intervention scores were significantly lower in the FCC group, with more pronounced differences in SAI scores (*P <* 0.001) Table-II.

**Table-II:** Pre-procedure anxiety scores (*χ̅*±*S*).

*Group*	n	SAI score		TAI score	
		Pre-nursing	Post-nursing	Pre-nursing	Post-nursing
FCC	90	57.79±4.75	43.86±5.53	50.06±5.19	42.78±4.48
Standard care	90	57.82±4.81	51.25±4.66	50.05±5.22	45.27±4.54
t-value		0.042	9.695	0.013	3.704
P-value		0.966	<0.001	0.990	<0.001

***Note:*** SAI - State Anxiety Inventory; TAI - Trait Anxiety Inventory.

Increases in SBP, DBP and HR after the procedure were significantly greater in the standard care group than in the FCC group (all *P <* 0.001) Table-III. Parental knowledge scores for medication administration, dietary management, complication prevention and monitoring of disease progression were significantly higher in the FCC group (all *P <* 0.001) Table-IV.

**Table-III:** Physiological stress response indicators in pediatric patients (*χ̅*±*S*).

Group	n	SBP (mmHg)		DBP (mmHg)		HR (beats/min)	
		Pre-procedure	Post-procedure	Pre-procedure	Post-procedure	Pre-procedure	Post-procedure
FCC	90	100.05±9.58	102.68±7.55	58.75±2.93	59.92±2.45	93.17±9.33	94.55±8.77
Standard care	90	100.69±9.49	109.75±9.62	58.69±2.85	64.78±2.52	92.86±9.29	99.35±9.32
t-value		0.450	5.485	0.139	13.118	0.223	3.558
P-value		0.653	<0.001	0.889	<0.001	0.824	<0.001

***Note:*** SBP - systolic blood pressure; DBP - diastolic blood pressure; HR - heart rate.

**Table-IV:** Parental knowledge scores across four caregiving domains (*χ̅*±*S*).

Group	n	Medication administration		Dietary management		Complication prevention		Monitoring of disease progression	
		Pre-nursing	Post-nursing	Pre-nursing	Post-nursing	Pre-nursing	Post-nursing	Pre-nursing	Post-nursing
FCC	90	55.89±5.32	83.86±7.93	52.48±6.11	88.39±7.36	44.60±5.52	82.17±7.21	54.51±5.75	82.19±7.38
Standard care	90	56.37±5.49	69.33±6.76	53.02±6.08	79.40±6.58	44.79±5.62	73.43±6.76	54.61±5.63	73.54±6.32
t-value		0.596	13.228	0.594	8.639	0.229	8.389	0.119	8.534
P-value		At 1-month post-procedure	<0.001	0.553	<0.001	0.819	<0.001	0.905	<0.001

At the one-month follow-up, the FCC group demonstrated superior outcomes in all three evaluated parameters. The satisfaction rate among children and caregivers was significantly higher in the FCC group, while both the incidence of adverse events and the recurrence rate of gastrointestinal symptoms were significantly lower compared with the standard care group. All differences were statistically significant (all *P <* 0.001) Table-V.

**Table-V:** Comparison of satisfaction, adverse events and symptom recurrence between the FCC and standard care groups (n[%]).

*Group*	n	Satisfaction rate among children and caregivers	Incidence of adverse events	Recurrence rate of gastrointestinal symptoms
FCC	90	86(95.56)	2(2.22)	2(2.22)
Standard care	90	68(75.56)	6(6.66)	4(4.44)
χ^2^ value		14.565	14.400	13.240
P-value		<0.001	<0.001	<0.001

## DISCUSSION

This study demonstrated that, compared with the standard care group, children in the FCC group exhibited significantly better procedural cooperation and a marked reduction in total procedural duration. A previous study^9^ reported that the structured educational module, which employed 3D anatomical animations and systematic explanations of Hp pathogenesis, was effective in reshaping perceptions of invasive diagnosis and treatment among pediatric patients and their parents. The study by McCarthy E et al.^10^ indicated that cognitive distortions account for approximately 60% of procedural fear in pediatric patients and that the caregiver’s level of medical knowledge significantly influences the child’s emotional regulation.

In terms of intervention techniques, the FCC group benefited from hands-on training using a gastroscopy simulation model, particularly the “three-point fixation method” for positioning. This specialized training improved caregivers’ ability to manage patient positioning, thereby reducing the need for repeated intraoperative adjustments. Additionally, the “three-step soothing strategy,” implemented through scenario-based learning effectively lowered children’s procedural resistance.^11^ These results align closely with findings from Deribe L et al.^12^, who reported that FCC significantly alleviated psychological distress in pediatric oncology patients. Together, these data support the conclusion that FCC is a robust model for reducing anxiety in pediatric care settings.

This study further revealed that following the intervention, children in the FCC group exhibited significantly lower SAI scores and reduced fluctuations in physiological stress responses. Specifically, caregiver-administered soothing techniques (*e.g*., tactile stimulation via stress-relief balls) are believed to activate cutaneous mechanoreceptors, thereby modulating the hypothalamic-pituitary-adrenal axis and reducing excessive cortisol secretion.^13^,^14^ Additionally, positive reinforcement strategies (e.g., encouragement badges) were found to promote parasympathetic nervous system dominance, effectively counteracting the sympathetic arousal typically triggered by invasive medical procedures.^15^,^16^ Notably, the improvement in SAI scores was markedly greater than that in TAI scores, indicating that FCC is particularly effective in mitigating situational anxiety, which is especially relevant in procedural settings.

Moreover, this study showed that parents in the FCC group achieved significantly higher scores in caregiving knowledge, which translated into tangible clinical benefits: lower incidence rates of adverse events and symptom recurrence within one-month post-procedure. Evidence suggests that FCC training modules are highly absorbable by caregivers. For example, emergency drills such as the “golden 30-minute” response to vomiting enhanced parents’ ability to recognize early risk signs, allowing for timely intervention in complications like aspiration.^17^,^18^ Additionally, personalized recovery protocols, including weight-based liquid dietary plans, proved effective in addressing adherence challenges related to pediatric nutritional intake. The day three follow-up call served as a critical checkpoint for evaluating symptom progression, thereby enhancing the precision of home-based recovery monitoring.^19^,^20^

### Limitations:

Despite its promising findings, this study has several limitations. Notably, it did not assess differential responses to the FCC across various pediatric age groups (*e.g*., toddlers *vs*. school-aged children). Additionally, since comprehension and skill acquisition are often correlated with educational attainment, the development of tiered or adaptive educational strategies is needed to ensure equitable benefit across diverse caregiver populations. In view of this, further improvements will be made in future research to make more scientific research results.

## CONCLUSIONS

The FCC model can significantly enhance procedural cooperation and safety in pediatric patients undergoing gastroscopy, effectively reduce anxiety and physiological stress responses, strengthen caregiver competencies and improve longer-term clinical outcomes. These findings support the clinical value and broad applicability of FCC.

### Authors’ Contributions:

**TM:** Contribution in conception, design of study, acquisition and interpretation of data, drafting of manuscript, research coordination and management, and is responsible and accountable for the accuracy or integrity of the work.

**XS:** Conception and design, drafting of manuscript, revising and editing the manuscript.

**CZ:** Drafting, editing and revising the manuscript.

**DT** and **GZ:** Conception and design of study, drafting of manuscript, revising and editing the manuscript research coordination and management.

All authors have read and approved the final manuscript.
